# The Binding Ring Illusion: assimilation affects the perceived size of a circular array

**DOI:** 10.12688/f1000research.2-58.v2

**Published:** 2013-04-25

**Authors:** J Daniel McCarthy, Colin Kupitz, Gideon P Caplovitz

**Affiliations:** 1 Department of Psychology, University of Nevada Reno, Reno, NV, 89557, USA

## Abstract

Our perception of an object’s size arises from the integration of multiple sources of visual information including retinal size, perceived distance and its size relative to other objects in the visual field. This constructive process is revealed through a number of classic size illusions such as the Delboeuf Illusion, the Ebbinghaus Illusion and others illustrating size constancy. Here we present a novel variant of the Delbouef and Ebbinghaus size illusions that we have named the Binding Ring Illusion. The illusion is such that the perceived size of a circular array of elements is underestimated when superimposed by a circular contour – a binding ring – and overestimated when the binding ring slightly exceeds the overall size of the array. Here we characterize the stimulus conditions that lead to the illusion, and the perceptual principles that underlie it. Our findings indicate that the perceived size of an array is susceptible to the assimilation of an explicitly defined superimposed contour. Our results also indicate that the assimilation process takes place at a relatively high level in the visual processing stream, after different spatial frequencies have been integrated and global shape has been constructed. We hypothesize that the Binding Ring Illusion arises due to the fact that the size of an array of elements is not explicitly defined and therefore can be influenced (through a process of assimilation) by the presence of a superimposed object that does have an explicit size.

## Introduction

**Figure 1.**
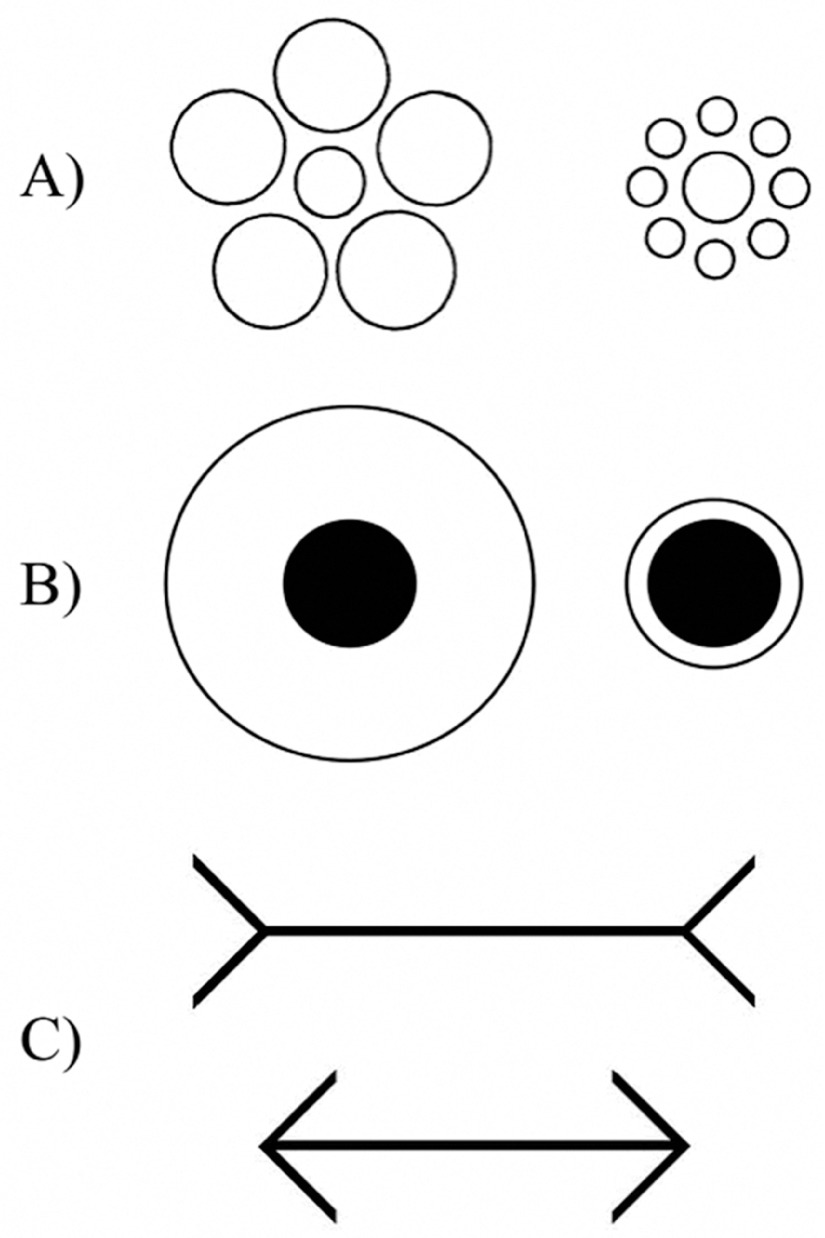
Notable size illusions: **A**) Ebbinghaus illusion, **B**) Delboeuf illusion, **C**) Müller-Lyer illusion.

Correctly perceiving the size of objects is essential to successfully interact with the world around us. Due to the fact that we sense the 3D visual world through an analysis of 2D retinal images, the process of size perception is intrinsically ambiguous. As a result, the perception of an object’s size arises from the integration of multiple sources of visual information including the size of its retinal image, its perceived distance ^1^, and its size relative to other objects in the visual scene ^2, 3^. These constructive processes are revealed through a number of classic size illusions such as the Ebbinghaus Illusion ^4^ ( Figure 1A), the Delboeuf Illusion ^5, 6^ ( Figure 1B), the Müller-Lyer Illusion ^7^ ( Figure 1C) and several others that illustrate how mechanisms that underlie size constancy sometimes lead to illusory percepts resulting from a discrepancy between retinal and perceived size. In each of these illusions, the perceived size of an explicitly defined object is influenced by the context in which it is presented. Most relevant to the current paper are the Delboeuf and Ebbinghaus illusions that demonstrate that the size of an inner circle is overestimated or underestimated depending on the surrounding context in which it is presented. Though several explanations have been proposed for these illusions, recent research demonstrates that the effect is largely determined by the relative size of the inducer(s), their distance from the target ^2^, and in the case of the Ebbinghaus Illusion, the completeness of the surrounding array of elements ^8^. Taken together, the balance of these factors determines the magnitude of the illusion and whether the inner circle is overestimated or underestimated.

**Figure 2.**
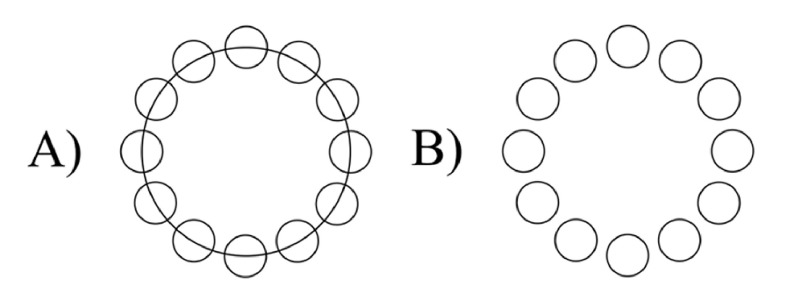
The Binding Ring Illusion. Which array of circles looks bigger? **A**) The test stimulus used in experiment 1 or **B**) The reference stimulus used in experiment 1.

Here we address the question of how we perceive the size of an implicitly defined object–an array of elements–by introducing a novel variant of the Ebbinghaus illusion that we have named the Binding Ring Illusion. We describe the illusion and investigate the underlying mechanisms that lead to misperceived size. A basic stimulus that elicits perception of the Binding Ring Illusion is composed of a circular array of small circles onto which a larger circle is superimposed as shown in Figure 2A. The superimposed circle leads to an underestimation of the perceived radius of the circular array relative to an equally sized array without the binding ring ( Figure 2B). To our knowledge, previous research on the Ebbinghaus Illusion has focused on the effect the surrounding elements have on the explicitly defined circle. Here we consider the possibility of mutual influence in that the inner circle may also lead to misperceived size of the surrounding array. In the following experiments, we investigate the magnitude of this illusory decrease in size and attempt to study the components of the stimulus that are responsible for the observed effect.

In experiment 1, we demonstrate that the effect of the binding ring can be quantified. In experiment 2 we demonstrate that the illusion arises due to assimilation toward the binding ring ^9, 10^. In experiment 3, we investigated the effect of spatial frequency. Finally, in experiments 4a and 4b we investigated the roles of local configural features in producing the illusion.

## General methods

### Participants

Five observers (3 men, 2 women; age range: 18–25; mean age = 20.2) participated in experiment 1, ten observers participated in experiment 2 (4 men, 6 women; age range: 19–28; mean age = 22.4), twelve observers participated in experiment 3 (6 men, 6 women; age range: 18–27; *M* = 21.75) and five observers participated in experiment 4 (3 men, 2 women; age range: 18–25; *M* = 20.6). All participants were naïve to the aims of the experiments, reported normal or corrected-to-normal vision and received course credit for their participation. Prior to participating, each observer provided informed consent according to the guidelines of the Department of Psychology and the IRB of the University of Nevada, Reno.

### Apparatus and display

Stimuli were presented on a Dell Trinitron P991 monitor (19 inches, 1024 × 768) with an 85 Hz refresh rate. The stimulus computer was a 2.4 GHz Mac Mini with an NVIDIA GeForce 320M graphics processor (256MB of DDR3 SDRAM). Stimuli were created and presented with the Psychophysics Toolbox ^11^ for MATLAB (Mathworks Inc., Natick, MA). In experiments 1, 2 and 4, the stimuli were white (120 cd/m ^2^) presented on a black (0.06 cd/m ^2^) background. In experiment 3, the stimuli were either low- or high-pass filtered and presented on a grey background (20.5 cd/m ^2^). Luminance values were measured with a Photo Research PR655 spectroradiometer. Participants placed their head on a chin rest and viewed the stimuli binocularly from a distance of 57cm.

## Experiment 1

The goal of experiment 1 was to establish a ‘standard’ configuration and quantify the magnitude of the illusion in order to serve as a baseline for further testing. Specifically, we sought to measure the perceived reduction in size of an array superimposed with a binding ring compared to an unbound array of equal size.

### Stimuli and procedures

**Figure 3.**
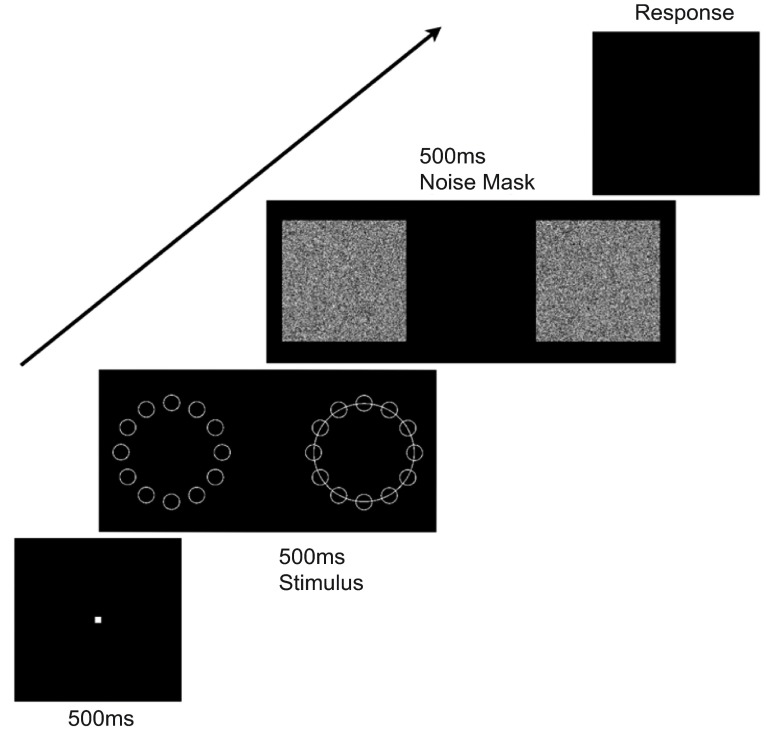
Schematic diagram of a trial in experiment 1. A fixation point appeared for 500ms followed by the presentation of the reference and test array for 500ms at which point the stimuli were removed from the screen and replaced by a noise mask for another 500ms to prevent the formation of afterimages. The screen then remained blank until participants indicated which array appeared larger via a key press.

The basic paradigm is illustrated in Figure 3. On a given trial, participants were presented with a small central fixation point (0.35º) for 500ms, followed by the additional simultaneous presentation of two circular arrays (a reference and a test) for 500ms at which point the stimuli were removed from the screen and replaced by a random noise mask displayed for 500ms to discourage afterimages. Participants indicated by pressing one of two buttons (two-alternative forced choice), which of the two stimuli had appeared larger. Each array consisted of 12 small equally spaced circles with radii of 0.05º visual angle. On every trial, the reference array had a fixed radius of 3º visual angle (from the center of the array to the center of any circular element). Using the method of constant stimuli ^12^, we investigated the perceived size of each of two test arrays compared to the reference array. The test array either had a binding ring superimposed or did not (the lack of a binding ring served as a control condition). In non-control conditions the binding ring had a radius selected to match the radius of the test circular array that was measured from the center of the array to the center of one of the smaller component circles. Because the control array did not have a superimposed binding ring it was identical to the reference array in all ways except its trial-by-trial size. As such, it was used to determine A) how accurately observers were able to perform the task (discriminate the sizes of the arrays) and B) to serve as a point of comparison for determining the size of the illusory effect. On each trial, the radius of the test array was selected from the following list: 2.5º, 2.6º, 2.8º, 2.9º, 3.0º, 3.1º, 3.3º, 3.4º and 3.5º.

On each trial, the centers of the two arrays were randomly positioned within a circular radius of 1.16º of visual angle centered 6.75º along the horizontal axis to the left or right of the central fixation point. This positional-jitter was used to prevent observers from basing their judgments on horizontal matching or distance comparisons with the edges of the monitor. Participants were instructed to maintain fixation on the center of the screen throughout the experiment. The sides on which the two arrays were presented were randomly determined on each trial. In total there were 18 trial types: nine test radii for both the test and control array types. Trials were pseudo-randomly ordered such that 20 of each trial type were presented in random order for a total of 360 trials. Prior to the experiment, participants were trained on 20 trials of the largest and smallest test array sizes that were not included in the analyses.

### Results

For each array size, we computed the percentage of times the test or control array was perceived to be larger than the reference. Thus, for the test (bound) and control (unbound) arrays, nine values (one for each radius) were calculated. Because the 2AFC task has two categorical responses, the following sigmoidal-shaped binomial-logit function was then fit to the corresponding data for each of the two test arrays using the MATLAB (glmfit() command) ^13^:

$$f(x)=100\times \left[\frac{e^{b_{1}+xb_{2}}}{1+e^{b_{1}+xb_{2}}}\right]$$

**Figure 4.**
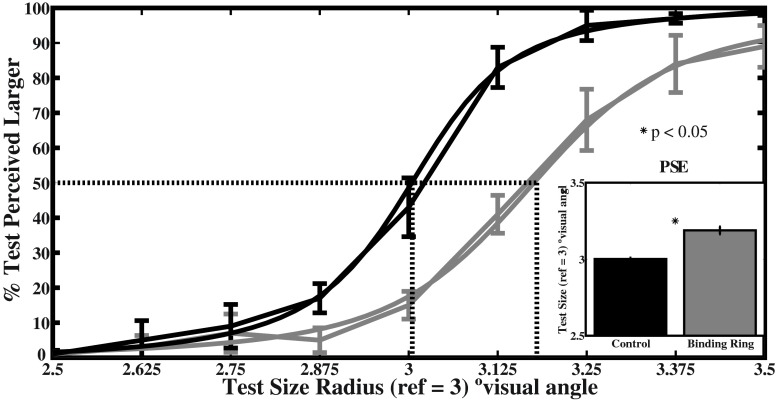
Results of experiment 1. Psychometric curves indicate the mean fit of the data averaged across all participants. The inset of the figure illustrates the mean points of subjective equality (PSEs) for the test and reference stimuli. The black curve indicates that participants were accurately judging the radius of an unbound array. The grey curve shows that the array radius must increase by ~0.18º to be perceived as being the same size as an unbound array. Thick curves with error bars indicate the mean response across participants for each array radius. Thin curves indicate the function fitted to the data. Error bars represent standard error of the mean PSE computed across subject.

The points of subjective equality (PSE) were determined for each subject by interpolating the 50% chance level from the function fit to the data ( *x* = – *b*_1_/ *b*_2_). The PSE indicates the size the test array needs to be in order to be perceived as equal in size to the reference. The resulting curves plotted for the mean responses across participants are shown in Figure 4. The clear and steep-sloped sigmoidal shaped psychometric curve derived from the control condition in which neither the reference nor control array have a binding ring confirm that participants were able to perform the task and accurately report their perceptions. Specifically, participants were at chance performance when the two arrays were indeed the same size. The rightward shift of the other psychometric curve, derived from the test (binding ring) condition, demonstrates that the size of the array was underestimated when the binding ring was present. The inset of Figure 4 illustrates the mean points of subjective equality across subjects for the test and control arrays.

A paired t-test between the PSEs of the test and control arrays revealed that the addition of the binding ring significantly ( *t*(4) = 7.71, *p* < 0.01) reduced the perceived size of the test array. The mean difference of the PSEs between the test and control arrays was ~ 0.18º of visual angle or 6% of the overall radius of the array. Thus, an array superimposed with a binding ring of radius 3.18º was perceived as having the same size as a no-binding ring array with a radius of 3º. This is comparable in magnitude to underestimation effects observed in the classic Ebbinghaus and Delbeouf Illusions ^14, 15^.

Experiment 1: Proportion of participants perceiving test arrays as being larger than reference arrays The first five rows of data indicate the proportion of times participants perceived an unbound test array as being larger than a fixed unbound reference array. The second five rows indicate the proportion of times participants perceived a binding ring array as being larger than a fixed unbound reference array. Each proportion was calculated from 20 trials.

## Experiment 2

**Figure 5.**
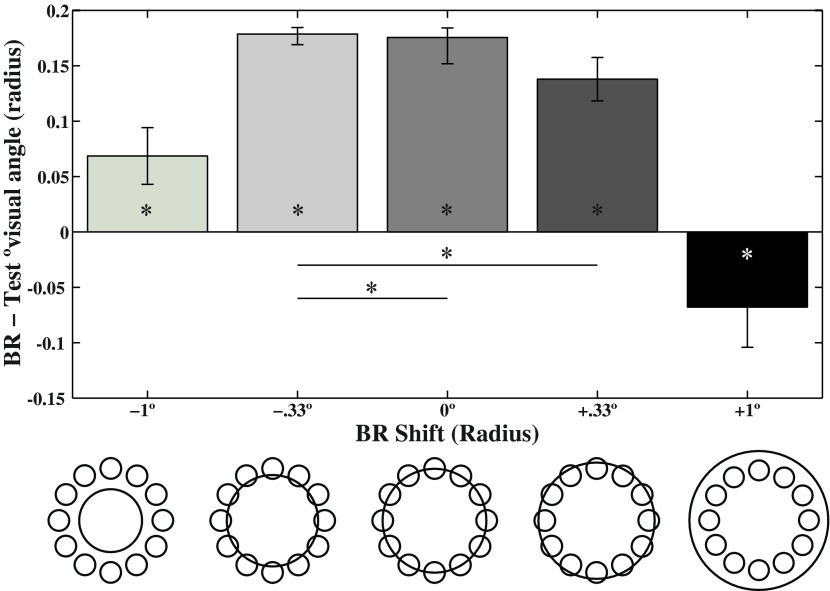
Stimuli and results of experiment 2. The radius of the binding rings from left to right: 2º, 2.67º, 3º, 3.33º and 4º; circular array radius was consistently 3º. The graph illustrates the PSEs for each stimulus type: lines and asterisks below the bars indicate significant (p < 0.05) differences, based on post-hoc paired t-tests, between the three overlapping binding ring conditions. Superimposed asterisks indicate significant changes in perceived size induced by the presence of the binding ring compared to an array without a binding ring (observed for all five conditions). A positive shift on the ordinate axis indicates that the array must increase in size to be perceived as having the same radius as the reference and vice versa. Error bars indicate standard error of the mean.

Having quantified the magnitude of the illusion in experiment 1, we explored possible mechanisms contributing to the underestimation of perceived size. Size illusions such as the Delboeuf and Ebbinghaus illusions have been explained on the basis of size assimilation (when the size of the element of interest is biased toward the reference component) and contrast (when the size of the element of interest is biased away from the reference component) ^9, 10, 16, 17^. Because the size of an array is not explicitly defined, it remains unclear whether the illusion arises due to assimilation or contrast with the binding ring. In order to determine if the illusion is mediated by assimilation or contrast we investigated the effect of changing the size of the binding ring (bottom of Figure 5). If assimilation is responsible for the effect, we would predict that the magnitude of the illusion should increase as the binding ring gets *smaller*. Alternatively, if the illusion arises due to contrast, the magnitude of the illusion should increase as the binding ring gets *larger*.

### Stimuli and procedures

The basic stimuli were the same as those used in experiment 1; however, due to the larger number of configurations tested, we used staircase procedures ^18^ rather than the method of constant stimuli. The individual trials within the staircases again consisted of a reference and test array simultaneously presented for 500ms. As in the previous experiment, the reference array had a fixed radius of 3º; however, unlike the previous experiment, the reference contained a binding ring and the test array did not. This was done so as to be able to compare the magnitude of the illusion for a fixed size array across binding-ring sizes. Separate staircases were run for five distinct reference conditions defined by the size of the binding ring with radii chosen from the following list: 2º, 2.67º, 3º, 3.33º and 4º (see Figure 5). Because the radius of each circular element was 0.5º, the binding ring did not physically overlap with the circular array in two of the five conditions and was either entirely inside (2º) or outside (4º) the array. Four staircases were run for each trial type–two in which the initial test or control array was larger than the reference array (descending) and two in which it was initially smaller (ascending). The starting radius for the test or control array was randomly selected to be 0.5º to 1º larger or smaller than the radius of the reference array. On each trial, participants completed a 2AFC task indicating which stimulus had appeared larger. According to standard staircase procedures, the size of the test array was adjusted by a step size ranging randomly on each iteration from 2 to 5 pixels (0.07º to 0.18º) in the direction opposite of the participant’s response. The staircase finished when four reversals were recorded. In total, each participant completed 20 staircases presented in pseudorandom order.

### Results

The mean of the four reversal points was calculated for each staircase and the PSE for each reference condition was obtained by averaging these results across the corresponding four staircases. Although we used a different experimental paradigm than in experiment 1, the size of the measured effect when the binding ring had a radius of 3.0º (same as in experiment 1) is comparable to that observed in experiment 1 (0.16º vs. 0.18º). The results illustrated on the top of Figure 5 indicate:

A) The size of the binding ring had a significant influence on the perceived size of the array (main effect of binding ring size-repeated measures ANOVA: *F*(4, 36) = 31.22, *p* < 0.001).

B) In each of the five conditions, the perceived size of the array was significantly influenced by the presence of the binding ring (one sample two-tailed, t-tests vs. zero: all p < 0.05 uncorrected).

C) The binding ring array was perceived as being smaller than an array without a binding ring in each of the four conditions in which the radius of the binding ring was less than the radius of the exterior portion of the array.

D) In the condition where the binding ring completely encompassed the array, the perceived size of the array was *larger* than when no binding ring was present. This is the reverse effect of the other four conditions.

E) The magnitude of the effect is greatest when the binding ring is superimposed on the array. A follow-up planned comparison of the three overlapping conditions with the two non-overlapping conditions revealed that the magnitude of the effect is significantly larger when the binding ring intersects the array compared to when it does not intersect the array ( *F*(1, 9) = 50.87, *p* < 0.001).

F) A second repeated measures ANOVA examining just the three superimposed conditions revealed that the size of the binding ring (within this range) significantly influences the magnitude of the illusion ( *F*(2, 18) = 3.84, *p* < 0.05). As can be seen in Figure 5, the magnitude of the illusion increases as the size of the binding ring is reduced for the three superimposed conditions. However, once the binding ring becomes too small, such that it no longer overlaps the array, the magnitude of the illusion is greatly decreased. That said, even in this innermost binding ring condition, the perceived size of the array is underestimated. It is noteworthy that this particular stimulus condition is quite similar to that typically used in the Ebbinghaus Illusion (see right side of Figure 1A). Here we demonstrate that there are in fact two illusory effects revealed in the Ebbinghaus Illusion, one operating on the inner circle (making it appear larger) and one operating on the outer array (making it appear smaller). Taken together, these observed effects are consistent with the assimilation hypothesis and inconsistent with the contrast hypothesis. Furthermore, these results appear to indicate that the outer edge of the circular array is serving as a boundary for the assimilation. When the radius of the binding ring exceeds this boundary, the assimilation bias is to increase the perceived size of the array. Similarly, if the radius of the binding ring is within this boundary, the assimilation bias is to decrease the perceived size of the array.

Experiment 2: reversal choices For subjects 1-10, values indicate the average difference in a radius at which participants made a reversal choice in the staircase for each of the five conditions tested labelled: BR Shift Xº. Each condition was tested 4 times and 5 reversals were recorded resulted in 20 total reversals. The 1st reversal of each trial was excluded from analysis. The mean for each subject(shown) was automatically computed by the stimulus presentation software: Adjustable_BR_Staircase.m on the basis of the remaining 4 reversals per trial. A copy of the program is available upon request.

## Experiment 3

**Figure 6.**
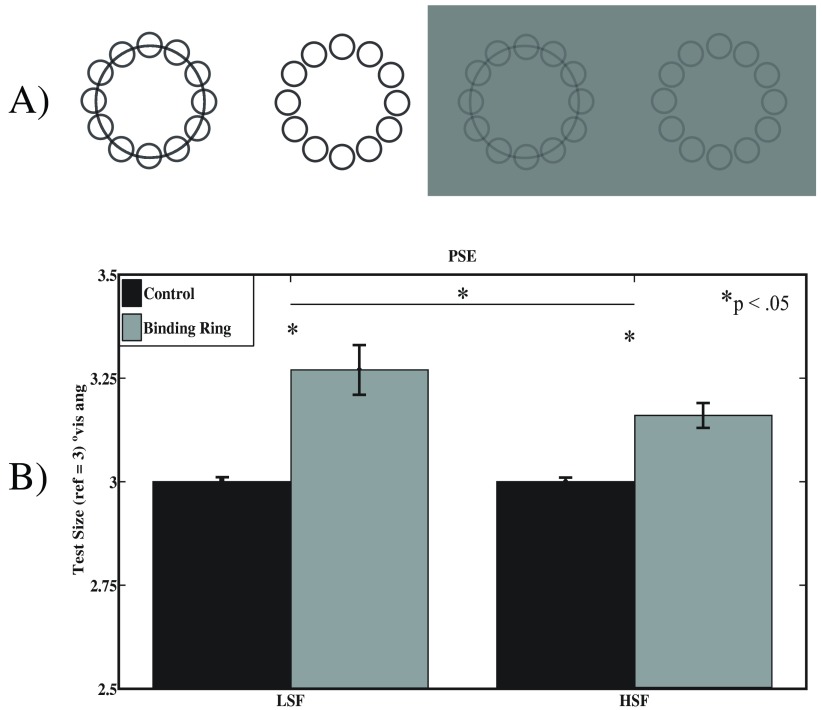
Spatial frequency manipulations. **A**) Low- (top left) and high-pass (top right) versions of the binding ring stimuli. **B**) Results of experiment 3. The points of subjective equality (PSE) of both high (HSF) and low spatial frequency (LSF) conditions are plotted for both the control and binding ring conditions. Error bars represent standard error of the mean. Asterisks indicate significance at p < 0.05.

In this and the following experiment, we attempt to identify specific stimulus factors that may influence the Binding Ring Illusion. It is suggested that some visual illusions, including the Müller-Lyer ^19^ and Oppel-Kundt ^20, 21^ or filled area illusion ^22^, are mediated by differential processing of low- and high-spatial frequency information ^23– 27^. Specifically, differential effects can be obtained resulting in changes to the magnitude of the illusions depending on spatial frequency filtering. Here, we investigated the effect of spatial frequency filtering on the magnitude of the Binding Ring Illusion ( Figure 6A).

### Stimuli and procedures

The stimuli and procedures used in experiment 3 were analogous to those used in experiment 1 except that the stimuli were either high- or low-pass filtered. The high-pass cutoff was set at (2 cpd) and the low-pass cutoff was (0.5 cpd). Stimuli were presented on a gray (20.5 cd/m ^2^) background. In each case, similarly filtered stimuli were compared to each other (i.e. a high spatial frequency (HSF) reference was compared to a HSF test or control). In total there were 36 trial types: nine test radii (same as in experiment 1) for the test and control array types (with and without binding ring) in both HSF and low spatial frequency (LSF) conditions. Trials were pseudorandomly ordered so that 20 of each trial type were presented for a total of 720 trials.

### Results

After fitting the curves using the same procedures described above, we conducted a 2 (binding ring vs. control) × 2 (HSF vs. LSF) repeated measures ANOVA on the derived PSE for each of the four conditions. This analysis revealed a main effect of the binding ring on perceived size ( *F*(1, 11) = 27.05, *p* < 0.01), a main effect of spatial frequency on perceived size ( *F*(1, 11) = 12.30, *p* < 0.01) and a significant interaction between the binding ring and spatial frequency ( *F*(1, 11) = 5.57, *p* < 0.05). As can be seen in Figure 6B, for both low- and high-pass stimuli, the array containing the binding ring was perceived to be smaller than when no binding ring is present. As reflected in the significant interaction, this effect is greater when the stimuli are low- as compared to high-pass filtered. Although the illusion was observed in both the LSF and HSF configurations, the size of the effect observed for the LSF condition (~0.3º) is substantially larger than that observed in the previous experiments that range from 0.16º to 0.18º. In contrast, when the stimuli were high-pass filtered, the resultant ~0.2º reduction in perceived size is comparable to that observed in the previous experiments.

Experiment 3: perceived size of test and reference arrays at high and low spatial frequency For the control conditions, the rows of data indicate the proportion of times participants perceived a unbound test array as being larger than a fixed unbound reference array. For the binding ring conditions, the rows of indicate the proportion of times participants perceived a binding ring array as being larger than a fixed unbound reference array. In the high spatial frequency (HSF condition), both arrays were high-pass filtered. In the low spatial frequency (LSF) condition, both arrays were low-passed filtered. Each proportion was calculated from 20 trials.

## Experiment 4a

Lastly we investigated the role of the local configural features of the stimulus. A number of visual illusions can be attributed to the processes of local configural features ^3, 8, 28^. In these two closely related experiments, we investigated the impact of presenting only parts of the binding ring on the presence or magnitude of the Binding Ring Illusion. In doing so we address the question of whether the Binding Ring Illusion is mediated by the processing of specific local features, or perhaps on the basis of more global representations of the objects present in the image.

**Figure 7.**
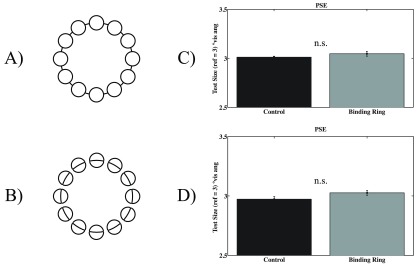
Configurally altered binding ring stimuli. **A**) The test stimulus used in experiment 4a. **B**) The test stimulus used in experiment 4b. **C**) The results of experiment 4a. **D**) The results of experiment 4b. There was no significant difference (n.s.) in perceived size between the control and binding ring tests in either condition.

In the first part of this experiment, the binding ring only connected the array of elements so that it was not visible in the interiors ( Figure 7A). In the second part of the experiment, the binding ring was present solely within the interiors of the array elements ( Figure 7B) leaving them unconnected from each other.

### Stimuli and procedures

The stimuli and procedures used in experiments 4a and 4b were identical to those used in experiment 1 except that in the test condition, the binding ring only connected the array of elements as if viewing a chain of pearls (experiment 4a) or the binding ring was only visible in the interiors of the array elements (experiment 4b).

### Results 4a

The data shown in Figure 7C were analyzed using the same curve fitting method described in the results of experiment 1. A paired samples t-test between the PSEs of the test and control arrays revealed no significant difference in perceived size ( *t*(4) = 2.05, *ns*).

### Results 4b

The data shown in Figure 7D were analyzed using the same curve fitting method described in the previous experiments. A paired t-test between the PSEs of the test and control arrays again revealed no significant difference in perceived size ( *t*(4) = 1.93, *ns*).

The Binding Ring Illusion was not observed in either of the partial binding ring configurations tested here. As such, we can conclude that processing of the preserved local features does not underlie the illusion. Alternatively, these results suggest that mechanisms within a higher, more global stage of processing may underlie the illusion.

Experiment 4: perceived size of test and reference arrays with lines only present either within the interior of the elements or connecting the elements In experiment 4a the first five rows of data indicate the proportion of times participants perceived an unbound test array as being larger than a fixed unbound reference array. The second five rows of indicate the proportion of times participants perceived an array with a line connecting the local elements as being larger than a fixed unbound reference array. Each proportion was calculated from 20 trials. In experiment 4b the first five rows of data indicate the proportion of times participants perceived an unbound test array as being larger than a fixed unbound reference array. The second five rows of indicate the proportion of times participants perceived an array with a line intersecting only the interiors of the elements as being larger than a fixed unbound reference array. Each proportion was calculated from 20 trials.

## General discussion

In this manuscript we introduced a new size illusion that we call the Binding Ring Illusion. The Binding Ring Illusion is a variant of the Delboeuf and Ebbinghaus Illusions and demonstrates that the perceived size of an array is subject to effects of assimilation. Specifically, the perceived size of a circular array of elements is underestimated when a ‘binding ring’ is superimposed on the array. The purpose of the above experiments was to demonstrate that the illusion can be quantified, to investigate possible explanations for its occurrence and to begin to characterize the stimulus factors that lead to misperceived size.

A number of observations can be made based on the results of these experiments. Firstly, the Binding Ring Illusion can indeed be quantified. Using both methods of constant stimuli and adaptive staircases we were able to measure significant differences in the perceived size of an array as a function of the presence and size of a binding ring. Secondly, the size of the binding ring significantly influences the magnitude of the illusion (experiment 2). Specifically, in order to produce the greatest effect, the binding ring has to superimpose the array and furthermore, as the size of the superimposed ring decreases, the magnitude of the illusion increases. These findings are consistent with existing assimilation theories of similar size illusions such as the Delboeuf and Ebbinghaus illusions ^9, 10, 15, 17^. In addition, the perceived size of the array was slightly *increased* only when the binding ring was large enough to completely encompass the array. This suggests that the outer radius of the array serves as the reference point for the assimilation process. It has been argued that assimilation is largely influenced by the perceived unification of the components as a single object ^17, 29^. This is consistent with our results such that the strongest assimilation effects occurred when the circle was superimposed on the array and could be easily perceived as a unified figure. In these conditions we observe a significantly larger magnitude of size illusion than when the binding ring was not superimposed on the array.

The Delboeuf and Ebbinghaus Illusions both demonstrate that the perceived size of an interior object can be influenced by the presence of a surrounding stimulus. The Binding Ring Illusion, on the other hand, provides a complimentary observation that the perceived size of a surrounding stimulus can be influenced by the presence of an interior stimulus. Indeed, the smallest binding ring condition in experiment 2 is an identical configuration to that commonly used to demonstrate the Ebbinghaus illusion.

Secondly, the magnitude of the illusion is greater when the stimuli are low- compared to high-pass filtered (experiment 3). However, in both cases the magnitude of the illusion is comparable if not greater than that observed with full spectrum stimuli. As such, we can conclude that: either processes within distinct spatial frequency channels can independently lead to the illusion, or the illusion is mediated by mechanisms at a later stage of processing that follows the integration of high- and low-spatial frequencies. In the latter case, one may conjecture that the initial LSF bias is attenuated once spatial frequency information has been integrated in object recognition areas such as those located in the inferotemporal cortex (IT) ^30^. This stands in contrast to several recent findings using functional and structural MRI that have implicated visual areas as early as V1 as playing a key role in the representation of perceived size ^31– 33^. Given the classical receptive field properties of V1 neurons, it is likely that these observations arise due to feedback to V1 from higher visual areas, that in the case of the Binding Ring Illusion may contain integrated representations of spatial frequency. This is in line with recent research on the Müller-Lyer Illusion using dynamic causal modelling ^34^. It was demonstrated that illusion strength could be predicted by modulating bilateral connections between the lateral occipital cortex (LOC) and right superior parietal cortex (SPC). The model suggests that LOC is involved in size scaling to generate size invariant object representations that are further processed in SPC and relayed back to V1 to generate conscious illusory percepts.

One hypothesis for why the illusion is greater in the LSF condition is that due to blur, the individual array elements in the LSF condition are perceptually larger than the HSF and full-spectrum conditions. Because the elements appear larger, the perceived distance between the outer radius of the array and the binding ring is increased. The results of experiment 2 suggest that this may lead to an increased assimilation effect. Alternatively, the blurring of the image increases the thickness of the binding ring and it could be the case that this increases its effect on the perceived size of the array. Further research will be necessary to fully determine why the blurred stimulus increased the magnitude of the illusion.

Thirdly, the illusion does not manifest when only the local configural features of the binding ring are present. This was true even when the binding ring only connects the array elements (experiment 4a). This result is intriguing because past work has demonstrated that elements that are perceptually grouped into a common object will be perceived to be closer together than those that are not. Specifically, if a series of dots are arranged to form a dotted contour, the distance between any two adjacent dots will be perceived as being shorter than the distance between any one of them and another equally-distanced dot that does not lie along the contour ^35^. One possible extension of this observation is that an object formed out of the grouping of individual elements may appear smaller on the basis of the elements appearing closer together. Based on this assumption we thought it possible that the partial binding ring of experiment 4a may serve as an additional cue that the individual elements belong to a common object and therefore lead to it appearing smaller. The configuration of experiment 4a explicitly links the elements of the array; however, this does not lead to the illusory reduction in perceived size. This may be explained by the observed effects of the Oppel-Kundt Illusion ^20, 21^ that demonstrates that the distance between two points is overestimated when it is filled with a number of tick marks compared to two equally spaced points of an undivided extent; however, as the density of these divisions increases, the effect diminishes ^36^. Therefore, connecting the interior elements should lead to a more veridical perception as observed here. As such, it is unlikely that the Binding Ring Illusion arises due to a misperception of the perceived distance between array elements.

## Conclusion

Although we readily perceive a circular array of elements as a circle, there are many possible alternate perceptions that could be formed. For example, the circles could be grouped into pairs symmetrical about the vertical axis, or perceived as ellipses arranged in an elliptical array that is receding in depth. That we perceive such a stimulus as a circle reflects the constructive processes that are embodied in the functional and structural architecture of the visual system. Importantly, the circle that we perceive does not explicitly exist in the retinal image and must therefore be constructed. As such, the size of the circle that we perceive must be itself constructed as well. The Binding Ring Illusion demonstrates that this constructive process includes the assimilation of other co-occurring stimuli, particularly those that spatially overlap the array.

## References

[1] Emmert E: Grossenverhaeltnisse der Nachbilder. *Klin Monbl Augenheilkd.* 1881;19:443–450

[2] Roberts B Harris MG Yates TA: The roles of inducer size and distance in the Ebbinghaus illusion (Titchener circles). *Perception.* 2005;34(7):847–856 10.1068/p5273 16124270

[3] Ross HE Plug C: The history of size constancy and size illusions. In: Walsh V, Kulikowski J, editors. Perceptual Constancy: Why Things Look the Way They Do. Cambridge: Cambridge University Press,1998;499–528 Reference Source

[4] Wundt W: Die geometrisch-optische Täuschungen [The geometric-optical illusions]. (Reprinted from Abhandlungen der mathematisch-physischen Klasse der Koniglich Sachsischen Gesellschaft der Wissenschaften). Leipzig: Teubner; Reference Source

[5] Delboeuf J: Seconde note sur de nouvelles illusions d'optique: Essai d'une théorie psychophysique de la manière dont l'oeil apprécie les grandeurs. Bulletins de l'Académie Royale des Sciences, Lettres et Beaux-arts de Belgique.1865; **20**:70–97 Reference Source

[6] Delboeuf JLR: Sur une nouvelle illusion d'optique. Academie Royale des Sciences, des Lettres et des Beaux Arts de Belgique. *Bulletins.* 1892;24:545–558

[7] Müller-Lyer FC: Optische urteilstäuschungen. *Archiv Anatomie Physiologie Physiologische Abteilung.* 1889;2:263–270 Reference Source

[8] Nemati F: Size and direction of distortion in geometric-optical illusions: Conciliation between the Müller-Lyer and Titchener configurations. *Perception.* 2009;38(11):1585–1600 10.1068/p6450 20120258

[9] Coren S Girgus JS: Seeing is deceiving: the psychology of visual illusions. Hillsdale: Lawrence Erlbaum Associates;1978;255 Reference Source

[10] Robinson JO: The psychology of visual illusion. London: Hutchinson,1972;288 Reference Source

[11] Brainard DH: The Psychophysics Toolbox. *Spat Vis.* 1997;10(4):433–436 10.1163/156856897X00357 9176952

[12] Laming D Laming J: F. Hegelmaier: on memory for the length of a line. *Psychol Res.* 1992;54(4):233–239 10.1007/BF01358261 1494608

[13] Wichmann FA Hill NJ: The psychometric function: I. Fitting, sampling, and goodness of fit. *Percept Psychophys.* 2001;63(8):1293–1313 10.3758/BF03194544 11800458

[14] Girgus JS Coren S Agdern M: The interrelationship between the Ebbinghaus and Delboeuf illusions. *J Exp Psychol.* 1972;95(2):453–455 10.1037/h0033606 5071920

[15] Oyama T: Japanese studies on the so-called geometrical-optical illusions. *Psychologia.* 1960;3:7–20 Reference Source

[16] Girgus JS Coren S: Assimilation and contrast illusions: differences in plasticity. *Percept Psychophys.* 1982;32(6):555–561 10.3758/BF03204210 7167354

[17] Goto T Uchiyama I Imai A: Assimilation and contrast in optical illusions. *Jpn Psychol Res.* 2007;49(1):33–44 10.1111/j.1468-5884.2007.00330.x

[18] Gescheider G: Chapter 3: The Classical Psychophysical Methods. In: Psychophysics: the fundamentals. 3rd ed. Hillsdale: Lawrence Erlbaum Associates;1997;45 Reference Source

[19] Carrasco M Figueroa JG Willen JD: A test of the spatial- frequency explanation of the Müller-Lyer illusion. *Perception.* 1986;15(5):553–562 10.1068/p150553 3588215

[20] Kundt A: Untersuchungen über Augenmass und optische Täuschungen. *Poggendorff Analle.* 1863;120:118–158 Reference Source

[21] Oppel J: Ueber geometrisch-optische Täuschungen (Zweite Nachlese). *Jahresber Phys Verein Frankfurt.* 1860/1861;1861:26–37 Reference Source

[22] Giora E Gori S: The perceptual expansion of a filled area depends on textural characteristics. *Vision Res.* 2010;50(23):2466–2475 10.1016/j.visres.2010.08.033 20801140

[23] Ginsburg AP: Psychological correlates of a model of the human visual system. DTIC Document,1971;119 Reference Source

[24] Ginsburg AP: Visual information processing based on spatial filters constrained by biological data. DITC Document,1978;338 Reference Source

[25] Ginsburg AP: Visual form perception based on biological filtering. Sensory experience, adaptation and perception.1984;53–72 Reference Source

[26] Ginsburg AP: Is the illusory triangle physical or imaginary? *Nature.* 1975;257(5523):219–220 10.1038/257219a0 1161021

[27] Ginsburg AP Evans DW: Predicting visual illusions from filtered images based upon biological data (A). *J Opt Soc Am.* 1979;69:1443 Reference Source

[28] Zöllner F: Ueber eine neue Art von Pseudoskopie und ihre Beziehungen zu den von Plateau und Oppel beschrieben Bewegungsphaenomenen. *Annalen Physik Chemie.* 1860;186(7):500–523 10.1002/andp.18601860712

[29] Goto T Uchiyama I Imai A: Assimilation and contrast. Handbook of the science of illusion.2005;164–176 Reference Source

[30] Kveraga K Ghuman AS Bar M: Top-down predictions in the cognitive brain. *Brain Cogn.* 2007;65(2):145–168 10.1016/j.bandc.2007.06.007 17923222 PMC2099308

[31] Murray SO Boyaci H Kersten D: The representation of perceived angular size in human primary visual cortex. *Nat Neurosci.* 2006;9(3):429–434 10.1038/nn1641 16462737

[32] Schwarzkopf DS Song C Rees G: The surface area of human V1 predicts the subjective experience of object size. *Nat Neurosci.* 2011;14(1):28–30 10.1038/nn.2706 21131954 PMC3012031

[33] Sperandio I Chouinard PA Goodale MA: Retinotopic activity in V1 reflects the perceived and not the retinal size of an afterimage. *Nat Neurosci.* 2012;15(4):540–542 10.1038/nn.3069 22406550

[34] Plewan T Weidner R Eickhoff SB: Ventral and dorsal stream interactions during the perception of the Muller-Lyer illusion: evidence derived from fMRI and dynamic causal modeling. *J Cogn Neurosci.* 2012;24(10):2015–2029 10.1162/jocn_a_00258 22721374

[35] Coren S Girgus JS: Principles of perceptual organization and spatial distortion: the gestalt illusions. *J Exp Psychol Hum Percept Perform.* 1980;6(3):404–412 10.1037/0096-1523.6.3.404 6447756

[36] Wackermann J Kastner K: Determinants of filled/empty optical illusion: Search for the locus of maximal effect. *Acta Neurobiol Exp (Wars).* 2010;70(4):423–434 21196950 10.55782/ane-2010-1814

