# Supplementary material for: The Binding Ring Illusion: assimilation affects the perceived size of a circular array
Source: F1000Res. 2013 Apr 25;2:58. Originally published 2013 Feb 22. [Version 2] doi: 10.12688/f1000research.2-58.v2 (PMC3901510; doi:10.12688/f1000research.2-58.v2)
Supplement: Peer review history

# Peer review: Referee response for version 2

**Published:** 2014-01-23

**Authors:** Dejan Todorović

**Affiliations:** 1 Laboratory of Experimental Psychology, Department of Psychology, University of Belgrade, Belgrade, Serbia

The authors have taken into account my comments in the revised text. I have no further comments.

I have read this submission. I believe that I have an appropriate level of expertise to confirm that it is of an acceptable scientific standard.

# Peer review: Referee response for version 2

**Published:** 2014-01-21

**Authors:** Ming Meng

**Affiliations:** 1 Department of Psychological Brain Sciences, Dartmouth College, Hanover, NH, USA

I have read this submission. I believe that I have an appropriate level of expertise to confirm that it is of an acceptable scientific standard.

# Peer review: Referee response for version 2

**Published:** 2014-01-20

**Authors:** Simone Gori

**Affiliations:** 1 Developmental and Cognitive Neuroscience Lab, Department of General Psychology, University of Padua, Padua, Italy

I approve this version, I think this paper was already interesting in its first version and I believe the Authors did improve it even further in this new version.

I have read this submission. I believe that I have an appropriate level of expertise to confirm that it is of an acceptable scientific standard.

# Peer review: Referee response for version 1

**Published:** 2013-04-03

**Authors:** Simone Gori

**Affiliations:** 1 Developmental and Cognitive Neuroscience Lab, Department of General Psychology, University of Padua, Padua, Italy

The presented manuscript is very interesting and well written. The experiments are very well conducted and it is a pleasure to read. The authors introduce a new size illusion and they investigate it using a very good methodology, providing clear results. I have some suggestions that in my opinion could improve the manuscript even more, but my general opinion is extremely positive.

Please see my suggestions below:

1. The introduction is really short, and it doesn't cover the topic of size illusion properly. Several other interesting examples of size illusions should be cited and discussed to provide a better understanding of the topic. Firstly, I would suggest discussing the literature on the illusions that are cited by the authors which are very similar to the one that they introduced here. For example, several interesting papers have been produced on The Ebbinghaus Illusion (which should maybe be called the Ebbinghaus-Titchener illusion, referring also to the 1902 Titchener book that made it popular) that maybe should be discussed (just few examples, without pretending to be complete at all: Roberts *et al*. 2005; Nemati, 2009; Doherty *et al.*, 2010; Schwarzkopf and Rees, 2013). The same can be said for the related Delboeuf illusion, which is suggested to be caused by the same brain mechanisms by Roberts *et al*. (2005). A few examples of recent works that may be worth checking are: Zanuttini and Daneyko (2010); Jaeger and Long (2007). Moreover, other interesting size illusions could be mentioned in the introduction, for example: The Muller-Lyer Illusion (Zeman *et al*. 2013; Plewan *et al*. 2012; Proulx and Green, 2011), the Oppel-Kundt Illusion (Wackermann, 2012a,b; Savazzi *et al*., 2012) and the related filled area illusion (Giora and Gori, 2011); the Breathing Light Illusion (Gori and Stubbs, 2006; Anstis *et al*. 2007; Gori *et al*. 2010), StarTrek Illusion (Qian and Petrov, 2012), Accordion Grating Illusion (Gori *et al*., 2011; 2013; Yazdanbakhsh and Gori, 2011) and the Fat Face Illusion (Thompson and Wilson, 2012; Sun *et al*. 2012), to name just a few.

2. Moreover, it would be nice if the authors could explain in more detail why studying this new illusion is relevant. In other words I would like to know which characteristics separate this new configuration from the ones already known. 

3. In the introduction of Experiment 3, it should be noted that the filled area illusion (Giora and Gori, 2010) is influenced by spatial frequency and a similar procedure to the one used in this study was employed. As both are size illusions, it would be interesting to discuss the similarities between the two.

4. When the authors state that several illusions are based on local configuration features, as well as citing the 1860’s work, they should also cite some new literature to provide more up to date references.

5. In the discussion, the difference between perceiving a difference and perceiving a group (Gori and Spillmann, 2010) could be interesting to discuss in relation to the last experiment.

6. The link with the Oppel-Kundt Illusion could be discussed in more detail, for example, by also referring to the work done by Wackermann.

7. In the discussion, some references to the potential brain mechanisms involved would be the icing on the cake in my opinion. Visual illusions are open windows on how the brain works and they are interesting tools to investigate it in a non-invasive manner. It would be nice to read about the hypothetical brain mechanisms underlying specific illusions.

**References**

Anstis S, Gori S, Wehrhahn C. Afterimages and the breathing light illusion. *Perception*. 2007; **36**(5):791-4.

Doherty MJ, Campbell NM, Tsuji H, Phillips WA. The Ebbinghaus illusion deceives adults but not young children. *Dev Sci*. 2010 Sep 1; **13**(5):714-21.

Giora E, Gori S. The perceptual expansion of a filled area depends on textural characteristics. *Vision Res*. 2010 Nov 23; **50**(23):2466-75.

Gori S, Giora E, Agostini T. Measuring the Breathing Light Illusion by means of induced simultaneous contrast. *Perception*. 2010; **39**(1):5-12.

Gori S, Spillmann L. Detection vs. grouping thresholds for elements differing in spacing, size and luminance. An alternative approach towards the psychophysics of Gestalten. *Vision Res*. 2010 Jun 11; **50**(12):1194-202.

Gori S, Stubbs DA. A new set of illusions--the dynamic luminance-gradient illusion and the breathing light illusion. *Perception*. 2006; **35**(11):1573-7.

Gori, S., Giora, E., Yazdanbakhsh, A. and Mingolla, E. (2011) ‘ A new motion illusion based on competition between two kinds of motion processing units: The Accordion Grating’, *Neural Networks*, **24**: 1082-1092.

Gori, S., Giora, E., Yazdanbakhsh, A. and Mingolla, E. (2013) ‘ The novelty of the “Accordion Grating Illusion”’, *Neural Networks*, 2013 Mar; **39**:52.

Jaeger T, Long S. Effects of contour proximity and lightness on Delboeuf illusions created by circumscribed letters. *Percept Mot Skills*. 2007 Aug; **105**(1):253-60.

Nemati F. Size and direction of distortion in geometric-optical illusions: conciliation between the Müller-Lyer and Titchener configurations. *Perception*. 2009; **38**(11):1585-1600.

Plewan T, Weidner R, Eickhoff SB, Fink GR. Ventral and dorsal stream interactions during the perception of the Müller-Lyer illusion: evidence derived from fMRI and dynamic causal modeling. *J Cogn Neurosci*. 2012 Oct; **24**(10):2015-29.

Proulx MJ, Green M. Does apparent size capture attention in visual search? Evidence from the Muller-Lyer illusion. *J Vis*. 2011 Nov 23; **11**(13).

Qian J, Petrov Y. StarTrek illusion--general object constancy phenomenon *?* *J Vis*. 2012 Feb 16; **12**(2):15. 

Roberts B, Harris MG, Yates TA. (2005). " The roles of inducer size and distance in the Ebbinghaus illusion (Titchener circles).". *Perception*. **34** (7): 847–56.

Savazzi S, Emanuele B, Scalf P, Beck D. Reaction times and perceptual adjustments are sensitive to the illusory distortion of space. *Exp Brain Res*. 2012 Apr; **218**(1):119-28.

Schwarzkopf DS, Rees G. Subjective Size Perception Depends on Central Visual Cortical Magnification in Human V1. *PLoS One*. 2013; **8**(3):e60550.

Sun YH, Ge L, Quinn PC, Wang Z, Xiao NG, Pascalis O, Tanaka J, Lee K. A new "fat face" illusion. *Perception*. 2012; **41**(1):117-20.

Thompson P, Wilson J. Why do most faces look thinner upside down? *Iperception*. 2012; **3**(10):765-74. 

Titchener, E.B. (1902). Experimental psychology: A manual of laboratory practice. New York, NY: MacMillan & Co., Ltd.

Wackermann J. Determinants of filled/empty optical illusion: Influence of luminance contrast and polarity. *Acta Neurobiol Exp (Wars)*. 2012; **72**(4):412-20.

Wackermann J. Determinants of filled/empty optical illusion: differential effects of patterning. *Acta Neurobiol Exp (Wars)*. 2012; **72**(1):89-94.

Yazdanbakhsh A, Gori S. Mathematical analysis of the accordion grating illusion: a differential geometry approach to introduce the 3D aperture problem. Neural Netw. 2011;24(10):1093-101.

Zanuttini L, Daneyko O. Illusory lightness in the Delboeuf figure. *Percept Mot Skills*. 2010 Dec; **111**(3):799-804.

Zeman A, Obst O, Brooks KR, Rich AN. The müller-lyer illusion in a computational model of biological object recognition. *PLoS One*. 2013; **8**(2):e56126.

I have read this submission. I believe that I have an appropriate level of expertise to confirm that it is of an acceptable scientific standard.

# Peer review: Referee response for version 1

**Published:** 2013-03-21

**Authors:** Dejan Todorović

**Affiliations:** 1 Laboratory of Experimental Psychology, Department of Psychology, University of Belgrade, Belgrade, Serbia

This paper is a study of a size perception illusion related to the Ebbinghaus illusion and the Delboeuf illusion. The study includes quantification of the basic phenomenon and some relevant parameters. It is well designed and executed, and contributes to our knowledge of this class of illusions. Size illusions of this class have been known for a long time and are still lacking a generally accepted explanation, but have not been studied much in recent years.

My only substantial criticism concerns the authors’ suggestion that the effect they studied is based on a high-level mechanism, late in the processing stream. I don’t find that they have offered enough evidence for such a conclusion. Note that recent research on the Ebbinghaus illusion ( Schwarzkopf *et al* 2010, *Nature Neuroscience*, **14**(1), 28-30), not cited by the authors, indicated that the strength of the illusion correlates with the size of V1, suggesting contributions to the illusion fairly early in the visual stream.

As a minor comment, it is commendable that the authors have cited the early research on size illusions, but some of the references, although often cited in that form in the recent literature, are in fact incorrect. For example, Oppel did not publish about the the illusion named after him in 1854/55 but in 1860/61, and in that paper he did not report about the dependence of the illusion on the number of tickmarks. For more details, see Wackermann J. & Kastner K. (2010), *Acta Neurobiol Exp*, **70**: 423–434. Also, Ebbinghaus did not publish the illusion named after him in the reference cited in the paper, and in fact seems to have never published it. For details, see Burton, G. (2001), *History of Psychology*, **4**, 228-244.

I have read this submission. I believe that I have an appropriate level of expertise to confirm that it is of an acceptable scientific standard.

# Peer review: Referee response for version 1

**Published:** 2013-03-18

**Authors:** Ming Meng

**Affiliations:** 1 Department of Psychological Brain Sciences, Dartmouth College, Hanover, NH, USA

The current paper presents a novel variant of visual size illusion that is named the Binding Ring Illusion by the authors. The illusory effect is quite strong as one could easily experience the illusion him/herself by looking at figure 2. The authors further tested several stimulus conditions to investigate why the Binding Ring Illusion occurs. Their results suggest that size assimilation at a relatively high level in the visual processing stream may underlie the illusion. I think this is a clearly written paper, the results are clear-cut, and the conclusion of this paper is well supported by the results.

I have read this submission. I believe that I have an appropriate level of expertise to confirm that it is of an acceptable scientific standard.
